# Association between serum uric acid and the risk of gestational diabetes mellitus: a multicenter cohort study

**DOI:** 10.3389/fnut.2026.1722321

**Published:** 2026-04-17

**Authors:** Qiong Li, Jiayi Zhou, Chenyang Zhao, Meng Li, Jingyang Li, Ying Gu, Chaoyan Yue

**Affiliations:** 1 Department of Obstetrics and Gynecology, The First People's Hospital of Chenzhou, Chenzhou, China; 2 Obstetrics & Gynecology Hospital of Fudan University, Shanghai Key Lab of Reproduction and Development, Shanghai Key Lab of Female Reproductive Endocrine Related Diseases, Shanghai, China; 3 Department of Obstetrics, Women's Hospital of Jiangnan University, Wuxi Maternal and Child Health Hospital, Wuxi, China

**Keywords:** cohort study, gestational diabetes mellitus, Mendelian randomization, multicenter cohort, uric acid

## Abstract

**Background:**

This study aimed to investigate the association between serum uric acid level in the before 20 weeks of gestation and gestational diabetes mellitus (GDM).

**Methods:**

A retrospective cohort study was conducted involving 44,609 singleton pregnant women from three medical centers from January 2018 to June 2024. The primary exposure was serum uric acid levels measured prior to 20 weeks of gestation. The main outcome of interest was GDM, with secondary outcomes including GDM requiring insulin therapy (GDMA2) and GDM complicated by pre-eclampsia (GDM&PE). Statistical methods such as smooth curve fitting, threshold effects, multivariate logistic regression, and subgroup analysis were employed to examine the relationship between uric acid and GDM. Additionally, a two-sample Mendelian randomization (MR) study was performed using genetic data from Genome-Wide Association Studies (GWAS) of serum uric acid and GDM.

**Results:**

The cohort study revealed a nonlinear relationship between uric acid level and GDM risk, with a turning point of 240 μmol/L. After adjusting for confounders, the odds ratio for GDM was 1.47(95% CI: 1.37-1.58) for uric acid level between 240 ~ 360 μmol/L compared to level below 240 μmol/L, the odds ratio for GDM was 2.60 (95% CI: 1.94-3.47) for uric acid levels ≥ 360 μmol/L. Similar positive associations were observed between uric acid level and GDMA2 and GDM & PE, which were consistent across subgroup analyses. The MR analysis indicated a causal relationship between uric acid and GDM (OR = 1.122, 95% CI: 1.016-1.239, *p* = 0.023).

**Conclusion:**

Elevated serum uric acid levels in the before 20 weeks of gestation are associated with an increased risk of GDM. Monitoring serum uric acid in early pregnancy helps in risk stratification for the management of individuals at high risk of GDM.

## Introduction

Gestational diabetes mellitus (GDM) is a common complication during pregnancy, with a prevalence rate of 14.0% reported in 2021 (1). GDM poses risks to both the mother and the child, including an increased likelihood of macrosomia, preterm labor, miscarriage, infections, cesarean section, fetal malformations, and long-term metabolic disorders (2, 3). Multiple factors influence the development of GDM, including diet, genetics, environmental factors, and lifestyle (3). A recent research has indicated that certain biochemical abnormalities presented in early pregnancy may correlate with the onset of GDM later in gestation (4). Therefore, identifying biomarkers for early prediction of GDM and conducting regular tests and early preventive measures in high-risk groups are crucial steps in reducing the incidence of GDM.

Uric acid (UA), the end product of purine nucleotide metabolism, has been linked to various diseases (5). Numerous studies have demonstrated that elevated level of UA increases the risk of metabolic conditions such as obesity, hypertension, hyperlipidemia, and diabetes (6–8). Given its simplicity and cost-effectiveness, UA measurement is hypothesized to serve as a potential early biomarker for predicting the onset of GDM. Despite growing interest in the relationship between UA and GDM, findings have been inconsistent (9–12). Observational studies may be subject to biases due to potential confounders and reverse causality, such as sample size, dietary factors, and duration of observation, making it difficult to definitively establish a relationship between UA and GDM.

This retrospective cohort study included a total of 44,609 singleton pregnant women from three medical institutions: Obstetrics and Gynecology Hospital of Fudan University, the First People's Hospital of Chenzhou, and Wuxi Maternal and Child Health Hospital. We utilized smooth curve fitting, threshold effect analysis, multivariate logistic regression, and subgroup analysis to examine the relationship between elevated serum UA level in the before 20 weeks of gestation and GDM. Additionally, a two-sample Mendelian randomization (MR) study was conducted to explore the causal relationship between UA and GDM using summary data from the Genome-Wide Association Study (GWAS) of serum UA and GDM. The combination of a multicenter pregnancy cohort study with MR analysis provided comprehensive evidence for the relationship between UA and GDM, minimizing the influence of confounding factors.

## Methods

**Figure 1:**
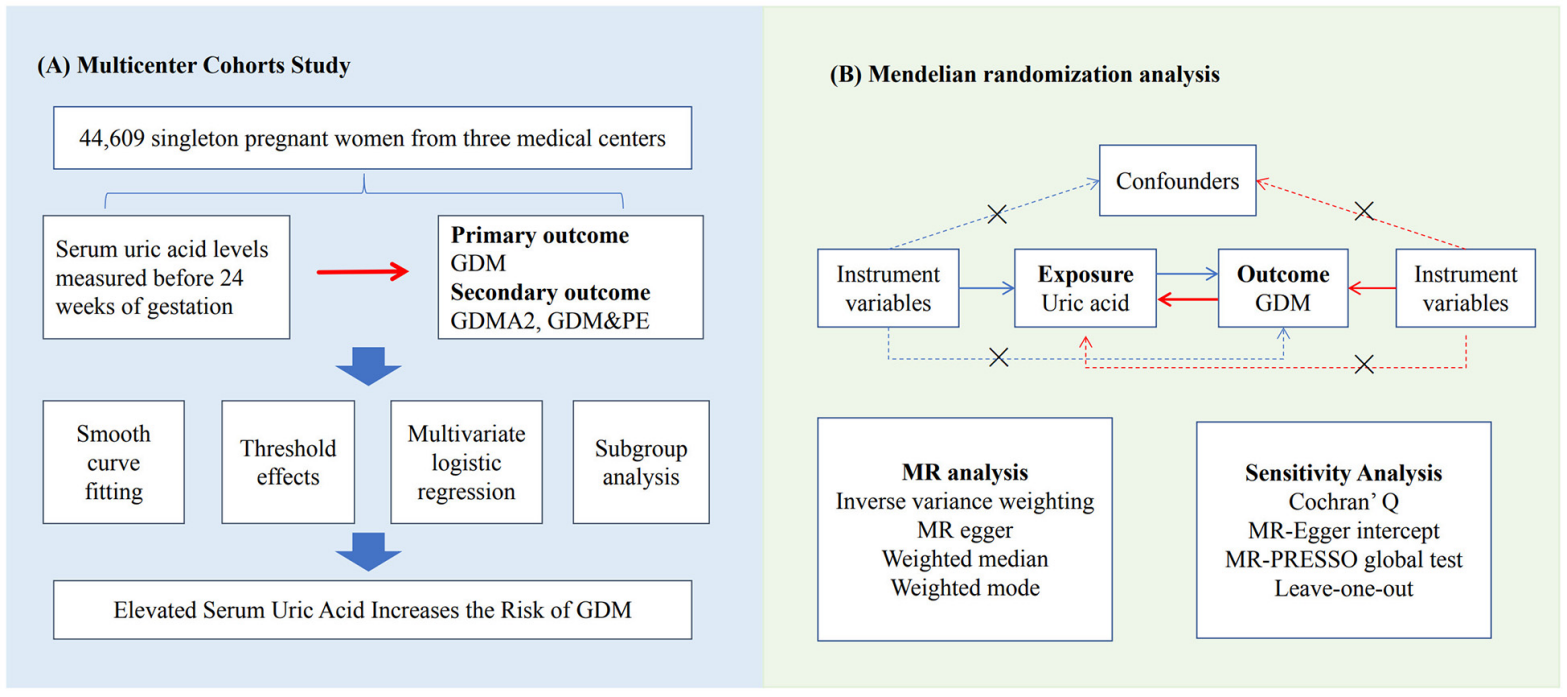
The overview of the study design. MR, Mendelian randomization; GDM, gestational diabetes mellitus; GDMA2, GDM requiring insulin therapy; GDM & PE, GDM combined with pre-eclampsia.

This study combines a multicenter retrospective cohort study based on real-world data with a Mendelian randomization study utilizing a public database. The investigation was divided into two phases: firstly, we analyzed the multicenter clinical data to investigate the linear and nonlinear relationships between serum UA level in the before 20 weeks of gestation and GDM and its associated outcomes using various statistical methods. Secondly, we evaluated the causal relationship between UA and GDM using the MR approach with publicly available data. The overview of the study design is shown in Figure 1.

### Retrospective study

#### Study population and design

This study included a total of 44,609 singleton pregnant women from January 2018 to June 2024 at three hospitals: Obstetrics and Gynecology Hospital of Fudan University, the First People's Hospital of Chenzhou, and Wuxi Maternal and Child Health Hospital. All singleton pregnant women who were documented at 11.59 ± 3.98 weeks of gestation and delivered at the respective healthcare institutions were included in our cohort. All clinical data were obtained from the hospital information system (HIS). Eligible participants were women who were at least 18 years of age, carrying a singleton pregnancy, and who received both antenatal care and delivered at one of the participating institutions. Women were excluded if serum uric acid was first measured after 20 weeks of gestation, had multiple gestations, a history of chronic hypertension, or pre-existing medical conditions including type 1 or type 2 diabetes, dyslipidemia, metabolic syndrome, renal or cardiovascular disease, or other significant chronic illnesses. Incomplete clinical or laboratory documentation also led to exclusion. A complete record required comprehensive maternal baseline data, including anthropometric measurements (height, prepregnancy weight), blood pressure, educational background, and medical history, as well as key perinatal outcomes such as gestational age at delivery and neonatal birth weight. The study was conducted in accordance with the ethical standards of the institutional review boards of the three participating hospitals and adhered to the tenets of the Declaration of Helsinki. Broad informed consent was obtained from all participants at the time of enrollment.

### Measurement of exposure

In this study, the primary exposure was the first serum UA measurement obtained during early pregnancy. Blood samples were collected from peripheral veins at the initial antenatal visit and analyzed using automated biochemical analyzers. In nonpregnant adults, hyperuricemia is typically defined as serum UA > 7.0 mg/dl in men and > 6.0 mg/dl in women. However, during pregnancy, UA levels decline by approximately 25%~35% due to increased renal clearance, with concentrations generally remaining below 4 mg/dl (1 mg/dl = 60 μmol/L) (13). Based on established thresholds and previous study (12, 13), hyperuricemia in women is commonly defined as UA > 360 μmol/L outside of pregnancy, whereas levels in healthy pregnant women usually stay under 240 μmol/L. Accordingly, UA was categorized into three groups: < 240 μmol/L, 240~359 μmol/L, and ≥360 μmol/L. Additionally, for dose-response analysis, UA was modeled as a continuous variable per 100 μmol/L increment in multivariable regression models.

### Assessment of outcome

The primary outcome measure in this study was GDM, which was categorized into GDM A1 and GDM A2. GDM A1 was defined as GDM managed with good glycemic control through diet alone, whereas GDM A2 referred to GDM requiring insulin therapy. The diagnostic criteria for GDM were based on the Oral 75g Glucose Tolerance Test (OGTT) recommended by the International Association for the Study of Diabetes and Pregnancy Group (IADPSG). Specifically, the 75g OGTT was administered between 24 and 28 weeks, and the blood glucose values at fasting, 1 h post-glucose intake, and 2 h post-glucose intake were required to be below 5.1 mmol/L, 10.0 mmol/L, and 8.5 mmol/L, respectively. GDM was diagnosed if any of these glucose measurements met or exceeded the specified thresholds (14).

The secondary outcomes included pharmacologically treated GDM (GDMA2) and GDM combined with pre-eclampsia (GDM & PE). PE was defined according to the guidelines of the American College of Obstetricians and Gynecologists as the presence of hypertension (systolic blood pressure ≥140 mmHg and/or diastolic blood pressure ≥ 90 mmHg) after 20 weeks of gestation, accompanied by proteinuria (≥ 300 mg of 24-h urinary protein), or the absence of proteinuria but accompanied by evidence of organ dysfunction (15). Data pertaining to these conditions were retrieved from HIS records.

### Assessment of covariables of interest

Drawing from prior evidence and clinical context (16, 17), a set of potential confounders was selected to assess their influence on the UA and outcome association. Covariates included maternal age (years), body mass index (BMI, kg/m^2^), tobacco and alcohol use, conception via *in vitro* fertilization (IVF), parity, adverse pregnancy history, family history of diabetes, test week, alanine transaminase (ALT, U/L), total cholesterol (TC, mmol/L), triglycerides (TG, mmol/L), fasting glucose (mmol/L) and creatinine. All variables were recorded at the first prenatal visit, with blood samples collected at a mean gestational age of 11.42 ± 3.84 weeks. Maternal age was classified as < 35 years (reference) and ≥35 years, while BMI was grouped as < 24 kg/m^2^ (underweight/normal) and ≥ 24 kg/m^2^ (overweight or obese).

### Statistical analysis

Continuous variables are presented as means with standard deviations (SDs), and categorical variables are presented as percentages. A four-step statistical analysis strategy was used to comprehensively evaluate the association between UA and GDM. First, participants were classified into the GDM and non-GDM groups according to clinical diagnostic criteria. Baseline characteristics were then compared between the two groups using the chi-square test for categorical variables and the independent-samples *t*–test for continuous variables. Second, to explore the potential nonlinear association between UA and GDM, smooth curve fitting and threshold effect analysis were performed using R software with the “rms” and “splines” packages. The optimal inflection point was identified by fitting a series of two-piecewise linear regression models across a pre-specified range of candidate knot values and selecting the knot with the maximum log-likelihood. Based on the identified threshold, a two-piecewise linear regression model was further applied to estimate the associations on either side of the inflection point, allowing for different slopes before and after the threshold. Nonlinearity was assessed by comparing the piecewise linear model with a conventional linear model using a likelihood ratio test. Third, after adjustment for the aforementioned confounding factors, multivariable predictive marginal proportions derived from the logistic regression model were used to estimate the adjusted odds ratios for the association between UA and GDM. Finally, subgroup analyses were conducted stratified by age, BMI, and parity, and interaction tests were performed to assess potential heterogeneity across subgroups. All tests were two-tailed, and a *P–*value < 0.05 was considered statistically significant. Statistical analyses were performed using IBM SPSS Statistics version 21.0 (IBM Corp., Armonk, NY, USA) and R software version 4.4.1 (R Foundation for Statistical Computing, Vienna, Austria).

### MR analysis

#### Data sources

A two-sample MR analysis was conducted to explore the potential causal relationship between UA and GDM using publicly available datasets from GWAS. Genetic data related to UA was obtained from the latest MRC IEU OpenGWAS dataset, which comprised 389,404 European participants (GWAS-ID: ebi-a-GCST 90014015) (18). Summary data for GDM were extracted from the FinnGen database, specifically the dataset with GWAS-ID finngen_R10_GEST_DIABETES, which includes 14,718 GDM cases and 215,592 healthy controls in European populations (19).

#### IV selection

The selection of instrumental variables (IVs) was guided by three fundamental assumptions (20). A general genome-wide significance threshold of *p* < 5 × 10^−8^ was established to filter variants. Subsequently, independent single nucleotide polymorphisms (SNPs) were obtained by setting the parameters *r*^2^ < 0.001 and clumping distance=10,000 kb. Only F-statistic > 10 being included in the analysis to mitigate the risk of weak instrumental bias. In addition, to mitigate the influence of BMI confounders on the results, SNPs significantly associated with BMI were also removed.

### Statistical analysis

The random-effects inverse variance weighted (IVW) method was employed as the primary MR analysis method to assess the causal relationship between UA and GDM. The MR-Egger, weighted median and weighted mode methods were used as complementary approaches to validate the accuracy and stability of the IVW results. The Cochran' *Q*-test was utilized to assess heterogeneity, with a *p*-value > 0.05 indicating no heterogeneity. The MR-Egger intercept and MR-PRESSO global test were applied to test for the presence of horizontal pleiotropy, with a *p*-value > 0.05 suggesting no horizontal pleiotropy. A leave-one-out sensitivity test was conducted by eliminating each SNP one by one to ascertain whether a specific SNP would impact the results. The MR analyses were carried out using the Two Sample MR software package and R Foundation version 4.3.0.

## Results

### Retrospective study

#### Baseline characteristics

Table 1 presents the baseline characteristics of the participants. Across the three healthcare institutions, a total of 38,248 participants were in the non-GDM group, and 6,361 were in the GDM group. Within the GDM group, there were 769 cases of GDMA2 and 527 cases of GDM & PE. Factors such as age, parity, BMI, family history of diabetes mellitus, alcohol, IVF, adverse pregnancy history, test week, ALT, TC, TG, creatinine, and fasting blood glucose showed statistically significant differences between GDM and controls (*p* < 0.05).

**Table 1:** Baseline characteristics of the study participants.

Characteristic	Control *n* = 38,248	GDM *n* = 6,361	Standardize diff.	*P-*value
Age (years)	31.12 ± 4.05	32.80 ± 4.29	0.40	< 0.001
BMI (kg/m^2^)	21.25 ± 2.90	22.98 ± 3.73	0.52	< 0.001
Test week	11.44 ± 3.80	11.58 ± 4.14	0.04	0.005
Uric acid (μmol/L)	214.40 ± 46.21	232.62 ± 54.90	0.36	< 0.001
Alanine transaminase (IU/L)	17.78 ± 15.51	20.43 ± 18.87	0.15	< 0.001
Total cholesterol (mmol/L)	4.56 ± 0.84	4.72 ± 0.83	0.19	< 0.001
Triglycerides (mmol/L)	1.34 ± 0.67	1.63 ± 0.89	0.38	< 0.001
Fast blood glucose (mmol/L)	4.47 ± 0.42	4.81 ± 0.78	0.54	< 0.001
Creatinine (μmol/L)	43.28 ± 6.56	42.97 ± 6.74	0.28	< 0.001
GDM A2				
No	21,103 (100.00%)	5,592 (87.91%)	0.52	< 0.001
Yes	1 (0.00%)	769 (12.09%)		
GDM & PE				
No	38,248 (100.00%)	5,834 (91.72%)	0.43	< 0.001
Yes	0 (0.00%)	527 (8.28%)		
Parity				
Primipara	27,364 (73.08%)	4,178 (67.24%)	0.13 (0.10, 0.15)	< 0.001
Multipara	10,078 (26.92%)	2,036 (32.76%)		
Family history of diabetes				
No	36,419 (95.24%)	5,629 (88.52%)	0.25 (0.22, 0.27)	< 0.001
Yes	1,822 (4.76%)	730 (11.48%)		
Tobacco				
No	32,953 (98.22%)	5,406 (98.06%)	0.01 (−0.02, 0.04)	0.413
Yes	598 (1.78%)	107 (1.94%)		
Alcohol				
No	32,199 (95.97%)	5,325 (96.59%)	0.03 (0.00, 0.06)	0.028
Yes	1,352 (4.03%)	188 (3.41%)		
IVF				
No	36,144 (94.50%)	5,833 (91.70%)	0.11 (0.08, 0.14)	< 0.001
Yes	2,104 (5.50%)	528 (8.30%)		
Adverse pregnancy history				
No	36,196 (94.64%)	5,890 (92.60%)	0.08 (0.06, 0.11)	< 0.001
Yes	2,052 (5.36%)	471 (7.40%)		

Mean ± SD for continuous variables; % for categorical variables.

BMI, body mass index; GDM, gestational diabetes mellitus; PE, preeclampsia, IVF, *In Vitro* fertilization.

### Detection of nonlinear relationships

**Figure 2:**
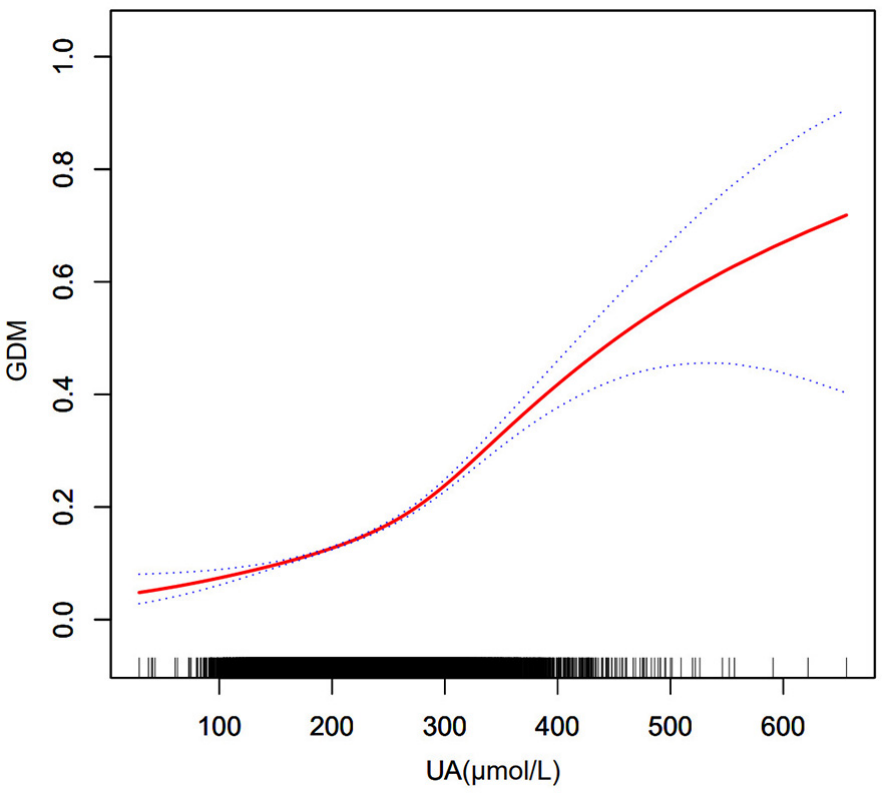
Smooth curve fitting of the association between serum UA levels and GDM. The red solid line represents the fitted relationship between UA and GDM, and the blue dashed lines indicate the 95% confidence intervals. Tick marks along the x-axis indicate the distribution of UA values. UA, uric acid; GDM, gestational diabetes mellitus.

Figure 2 illustrates the results from the smoothed curve fitting, indicating a nonlinear relationship between UA and GDM. The log-likelihood ratio test demonstrated a statistically significant difference between the linear and segmented linear regression models (*p* = 0.003). As shown in Table 2, the turning point **(K)** was 240 μmol/L. Below this point, the odds ratio (OR) was 1.8 (95% CI: 1.7-2.0, *p* < 0.001), while above it, the OR was 2.4 (95% CI: 2.1-2.6, *p* < 0.001), indicating that UA levels of 240 μmol/L or higher were associated with a significantly higher risk of GDM.

**Table 2:** Threshold effect analysis of the relationship between each 100 μmol/L increase in UA and GDM.

Models	Risk of GDM Adjusted OR (95%CI)	*P*-value
Model I		
One line slope	2.1 (2.0, 2.2)	< 0.001
Model II		
Turning point (K)	240 μmol/L	–
UA < 240 slope 1	1.8 (1.7, 2.0)	< 0.001
UA ≥ 240 slope 2	2.4 (2.1, 2.6)	< 0.001
Slope 2–Slope 1	1.3 (1.1, 1.5)	0.003
Predicted at 240	−1.7 (−1.8, −1.7)	–
P for log-likelihood ratio	–	0.003

Model I, linear analysis; Model II, non-linear analysis.

Adjust for age, parity, BMI, family history of diabetes, tobacco, alcohol, IVF, adverse pregnancy history, test week, alanine transaminase, total cholesterol, triglycerides, fast blood glucose and creatinine.

UA, uric acid; GDM, gestational diabetes mellitus; CI, confidence interval.

### The association between UA and GDM

Table 3 outlines that the associations between UA and GDM, GDMA2, and GDM & PE remained significant compared to the non-GDM group after fully adjusting for covariables of interest. Specifically, compared to UA < 240 μmol/L, the ORs for GDM, GDMA2, and GDM & PE were 1.47 (95% CI: 1.37–1.58), 1.47 (95% CI: 1.30–1.76), and 1.61 (95% CI: 1.30–1.99) for UA 240 ~ 360 μmol/L respectively. The ORs for GDM, GDMA2, and GDM & PE were 2.60 (95% CI: 1.94–3.47), 2.75 (95% CI: 1.70–4.47), and 4.05 (95% CI: 2.5–6.5) for UA≥360 μmol/L. Similar associations were observed for GDMA2 and GDM & PE when UA increased per 100 μmol/L.

**Table 3:** The independent effect of uric acid measures in before 20 weeks of gestation on the risk of primary and secondary outcomes.

Exposure	Model I		Model II	
	OR (95% CI)	*P*-value	OR (95% CI)	*P-*value
Primary outcome				
GDM				
< 240 (μmol/L)	Reference		Reference	
240~360 (μmol/L)	1.77 (1.67–1.87)	< 0.0001	1.47 (1.37, 1.58)	< 0.0001
≥360 (μmol/L)	4.55 (3.71–5.59)	< 0.0001	2.60 (1.94, 3.47)	< 0.0001
Continuous UA per 100 μmol/L	2.09 (1.98–2.20)	< 0.0001	1.72 (1.61, 1.85)	< 0.0001
Secondary outcome				
GDMA2				
< 240 (μmol/L)	Reference		Reference	
240~360 (μmol/L)	2.22 (1.91–2.58)	< 0.0001	1.47 (1.23, 1.76)	< 0.0001
≥360 (μmol/L)	8.81 (6.25–12.42)	< 0.0001	2.75 (1.70, 4.47)	< 0.0001
Continuous UA per 100 μmol/L	2.81 (2.49–3.17)	< 0.0001	1.78 (1.52, 2.10)	< 0.0001
GDM & PE				
< 240 (μmol/L)	Reference		Reference	
240~360 (μmol/L)	2.66 (2.22–3.18)	< 0.0001	1.61 (1.30, 1.99)	< 0.0001
≥360 (μmol/L)	15.80 (11.24–22.21)	< 0.0001	4.05 (2.52, 6.51)	< 0.0001
Continuous UA per 100 μmol/L	3.75 (3.28–4.28)	< 0.0001	2.05 (1.71, 2.46)	< 0.0001

Model I: None covariates were adjusted.

Model II: Age, parity, BMI, family history of diabetes, tobacco, alcohol, IVF, adverse pregnancy history, test week, alanine transaminase, total cholesterol, triglycerides, fast blood glucose and creatinine were adjusted.

UA, uric acid; GDM, gestational diabetes mellitus; PE, preeclampsia; OR, odds ratio; CI, confidence interval.

### Subgroup analysis

In the subgroup analyses stratified by age, BMI, and parity, a consistently positive associations between UA and GDM were observed across all subgroups. The results of the interaction tests were not statistically significant (*p* > 0.05 for interaction), suggesting that the adverse association between UA and GDM was not influenced by age, BMI, or parity (Table 4).

**Table 4:** Subgroup analysis for the association between UA and GDM.

GDM	Odds ratio (95% CI)	*P*-value	*P* for interaction	Adjusted odds ratio (95% CI)	*P-*value	*P* for interaction
Age (years)			0.3981			0.3981
Age ≤ 35						
< 240 (μmol/L)	Reference			Reference		
240~360 (μmol/L)	1.76 (1.65–1.88)	< 0.0001		1.42 (1.31, 1.55)	< 0.0001	0.350
≥360 (μmol/L)	4.91 (3.89–6.19)	< 0.0001		2.73 (1.97, 3.78)	< 0.0001	
Age > 35						
< 240 (μmol/L)	Reference			Reference		
240~360 (μmol/L)	1.83 (1.64–2.05)	< 0.0001		1.61 (1.39, 1.85)	< 0.0001	
≥360 (μmol/L)	3.68 (2.38–5.68)	< 0.0001		2.09 (1.12, 3.92)	0.0211	
BMI (kg/m^2^)			0.8051			0.737
BMI < 24						
< 240 (μmol/L)	Reference			Reference		
240~360 (μmol/L)	1.47 (1.37–1.58)	< 0.0001		1.47 (1.34, 1.60)	< 0.0001	
≥360 (μmol/L)	3.26 (2.31–4.61)	< 0.0001		2.42 (1.54, 3.82)	0.0001	
BMI ≥ 24						
< 240 (μmol/L)	Reference			Reference		
240~360 (μmol/L)	1.53 (1.38–1.70)	< 0.0001		1.45 (1.28, 1.66)	< 0.0001	
≥360 (μmol/L)	3.10 (2.33–4.12)	< 0.0001		2.84 (1.93, 4.18)	< 0.0001	
Parity			0.2864			0.983
Primipara						
< 240 (μmol/L)	Reference			Reference		
240~360 (μmol/L)	1.81 (1.69–1.94)	< 0.0001		1.45 (1.33, 1.58)	< 0.0001	
≥360 (μmol/L)	4.83 (3.77–6.19)	< 0.0001		2.51 (1.80, 3.50)	< 0.0001	
Multipara						
< 240 (μmol/L)	Reference			Reference		
240~360 (μmol/L)	1.65 (1.49–1.83)	< 0.0001		1.51 (1.32, 1.74)	< 0.0001	
≥360 (μmol/L)	4.06 (2.79–5.91)	< 0.0001		2.74 (1.50, 4.98)	0.0010	

Adjusted for age, parity, BMI, family history of diabetes, tobacco, alcohol, IVF, adverse pregnancy history, test week, alanine transaminase, total cholesterol, triglycerides, fast blood glucose and creatinine.

UA, uric acid; GDM, gestational diabetes mellitus; CI, confidence interval; BMI, body mass index.

### MR analysis

**Figure 3:**
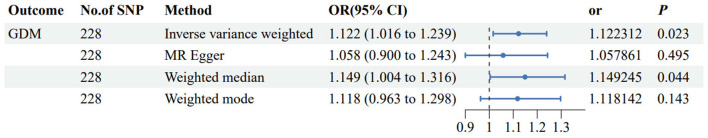
Forest plot to visualize the causal estimation of uric acid on gestational diabetes mellitus via Mendelian randomization analysis. No, number; IVW, inverse variance weighting; SNP, single nucleotide polymorphism; OR, odds ratio; CI, confidence interval; P, *P*-value.

We firstly identified 239 independent SNPs associated with UA. After eliminating 11 SNPs associated with the confounding factor BMI (including rs1045411, rs11835818, rs1229984, rs13107325, rs1446585, rs2439823, rs2540034, rs34811474, rs4441044, rs62106258, rs6857), we were left with 228 SNPs for the final MR analysis. The IVW method results indicated a causal relationship between genetically predicted UA levels and a higher risk of GDM (OR = 1.122, 95% CI 1.016–1.239, *p* = 0.023). Consistent results were obtained from MR-Egger, weighted median, and weighted mode methods (Figure 3). The MR scatter plot (Supplementary Figure S1) shows consistent directional effects of uric acid-associated SNPs on gestational diabetes mellitus, with no obvious outliers. The funnel plot (Supplementary Figure S2) displays symmetrical distribution of SNP-specific estimates, indicating minimal pleiotropy or publication bias. The Cochran' *Q*–test detected the presence of heterogeneity (*p* < 0.001). However, the MR-Egger intercept (*p* = 0.363) showed no evidence of horizontal pleiotropy (Supplementary Table S1). In the leave-one-out sensitivity analysis, no significant changes were observed in the overall MR estimates after individually removing instrument SNPs (Supplementary Figure S3). The reverse MR analysis revealed no significant association of GDM on UA (Supplementary Table S2).

## Discussion

This study is the first to investigate the association between UA level and GDM through a multicenter retrospective cohort study combined with MR analysis of publicly available GWAS data. The retrospective cohort study identified a nonlinear association between serum UA level and the risk of GDM, with the nature of these nonlinear associations varying based on UA level. Individuals exhibited a significantly increased odds ratio for GDM when UA level exceeded 240 μmol/L (OR = 1.47, 95% CI: 1.37–1.58). Robust sensitivity analyses confirmed the strength of the relationship between UA and GDM. MR analysis demonstrated that a 12.2% increase in the risk of GDM for every SD increase in UA level (OR = 1.122, 95% CI: 1.016–1.239), which further supported the causal association between UA and GDM.

Previous studies have been inconsistent regarding the precise relationship between UA and GDM. One study indicated that elevated serum UA were linked to an increased risk of GDM. Age-stratified analyses within this study showed that the association between UA and GDM was particularly pronounced in pregnant women aged 35 years and older (9). However, another study produced conflicting results, finding that elevated maternal blood uric acid levels between 16 and 18 weeks of gestation were associated with an increased risk of GDM, with women in the highest quartile showing a 55.7% increased risk compared to those in the lowest quartile. When these analyses were adjusted for age, similar results were observed only in pregnant women under 35 years of age, and this association was not evident in women over 35 years of age (10). Previous cohort studies have shown that elevated serum UA in early pregnancy is associated with an increased risk of subsequent GDM. In addition, among women with GDM, elevated UA has been associated with adverse pregnancy outcomes, including preterm delivery, macrosomia, premature rupture of membranes, and cesarean section (12, 21). These findings suggest that hyperuricemia may be a high-risk factor for GDM, though the optimal gestational week for measuring UA remains unclear. Additionally, since the development of GDM is influenced by multiple factors, further studies are necessary to control for confounding variables such as age and parity to provide more definitive evidence. Our study validated the relationship between UA level in the before 20 weeks of gestation and GDM through both observational and genetic evidence. Importantly, our methodology is less prone to the confounding factors, making the results more objective.

Serum UA level decrease significantly before the 20th week of normal pregnancy, attributed to increased glomerular filtration and reduced tubular reabsorption of UA (22). Elevated serum UA level during pregnancy may trigger adverse pathophysiological responses and increase the risk of adverse pregnancy outcomes (23). The impact of hyperuricemia on GDM likely involves multiple mechanisms. Firstly, elevated UA level affect insulin signaling, contributing to insulin resistance and endothelial dysfunction by reducing nitric oxide synthesis (24, 25). Animal studies have provided evidence suggesting that nitric oxide is an essential mediator of glucose uptake in muscle cells and adipocytes (26, 27). Additionally, hyperuricemia increases oxidative stress and the production of inflammatory cytokines, thereby exacerbating insulin resistance (28). UA also diminishes insulin secretion by inducing oxidative stress through the inhibition of the insulin gene (29). Case-control studies have shown that therapies aimed at lowering UA level, such as oral allopurinol, can improve insulin resistance caused by hyperuricemia (30, 31).

Serum UA, a metabolic byproduct of purine nucleotide metabolism, is a routinely measured, nutrition-related laboratory marker in early pregnancy. Emerging evidence suggests it may contribute to insulin resistance and metabolic dysfunction. Our study demonstrates that UA levels measured before 20 weeks of gestation are independently associated with an increased risk of GDM. As a simple and accessible test, early UA screening can facilitate risk stratification and early identification of high-risk women. This provides a critical window for timely intervention, particularly through targeted nutritional strategies such as reducing intake of purine-rich foods and added fructose, both of which are known to elevate UA levels. Implementing dietary modifications in response to elevated UA may help lower its concentration and potentially mitigate GDM risk. Therefore, integrating UA assessment with individualized nutritional counseling offers a practical and proactive approach in antenatal care, supporting early lifestyle guidance to prevent GDM onset and reduce the likelihood of adverse maternal and fetal outcomes.

The major strength of our study lies in the application of multiple statistical methods to analyze observational data and the validation of causality using MR analysis. The significant causal findings consistently offered robust evidence, even when accounting for the potential issues of large sample sizes and potential confounding factors. Furthermore, our analysis was a multicenter study utilizing clinical data from three regions, providing a larger and more representative sample size compared to single-center studies. However, our study also has limitations. There was heterogeneity in the results of the MR analysis, although a series of sensitivity analyses ensured the stability of the findings. Although our study identified the predictive value of UA level for GDM, it did not elucidate the underlying mechanisms by which UA affects GDM. Future research involving larger and more comprehensive studies is needed to address these issues.

## Conclusion

In conclusion, the concurrent analyses of observational and genetic data consistently demonstrated a relationship between elevated UA level and GDM. Measuring maternal serum UA level may serve as an important tool for predicting GDM, assisting clinicians in identifying pregnant women at high risk for GDM early on and providing preventive strategies, thereby reducing the risk of GDM and preventing adverse maternal and fetal outcomes.

## Data Availability

The datasets presented in this article are not readily available due to privacy protection. Requests to access the datasets should be directed to the corresponding author.
