# Supplementary material for: Association between serum uric acid and the risk of gestational diabetes mellitus: a multicenter cohort study
Source: Front Nutr. 2026 Apr 17;13:1722321. doi: 10.3389/fnut.2026.1722321 (PMC13133086; doi:10.3389/fnut.2026.1722321)
Supplement: Supplementary file 1 [file Figure_1.pdf]

Supplementary Figures

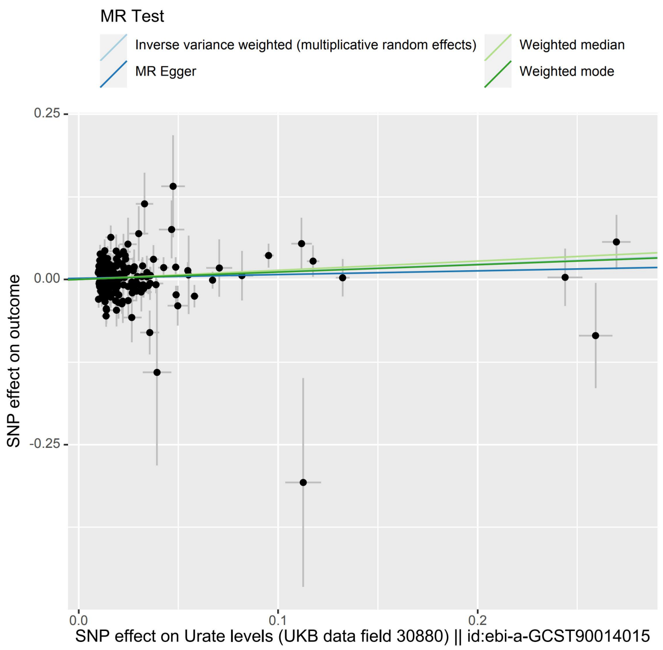

Figure S1. Scatter plot to visualize the causal effect of uric acid levels on gestational diabetes mellitus.

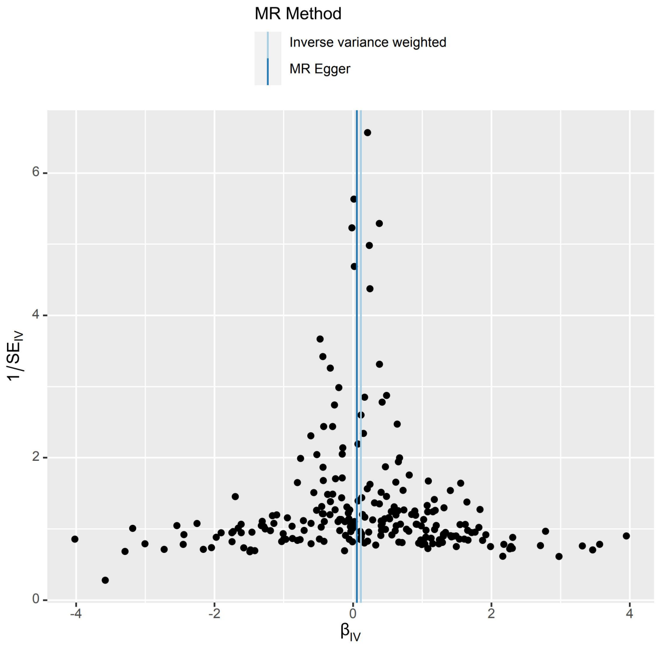

Figure S2. Funnel plot to visualize the causal effect of uric acid levels on gestational diabetes mellitus.

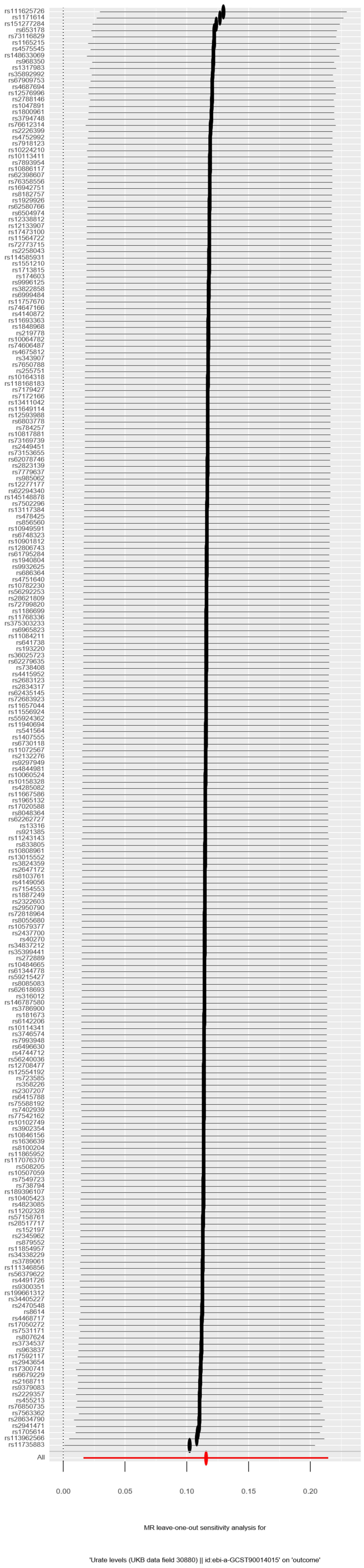

Figure S3. Leave-one-out inverse-variance weighted mendelian randomization analyses of uric acid levels on gestational diabetes mellitus.
