# Supplementary material for: Association between serum uric acid and the risk of gestational diabetes mellitus: a multicenter cohort study
Source: Front Nutr. 2026 Apr 17;13:1722321. doi: 10.3389/fnut.2026.1722321 (PMC13133086; doi:10.3389/fnut.2026.1722321)
Supplement: Supplementary file 2 [file Table_1.doc]

**Table S1** Sensitivity analysis of uric acid levels on gestational diabetes mellitus.

| UA levels | Heterogeneity | | Pleiotropy | |
| --- | --- | --- | --- | --- |
|  | Q of IVW | *P* value of IVW | *P* value of MR-Egger Intercept | *P* value of MR-PRESSO Global Test |
| GDM | 389.99 | <0.001 | 0.363 | 0.023 |

UA, uric acid; GDM, gestational diabetes mellitus; CI, confidence interval; MR, Mendelian randomization; IVW, inverse variance weighting.

**Table S2** The reverse MR analysis of causal associations of gestational diabetes mellitus with uric acid levels.

| GDM | IVW | | MR-Egger | | Weight Median | | Weight Mode | |
| --- | --- | --- | --- | --- | --- | --- | --- | --- |
|  | OR(95%CI) | *P* | OR(95%CI) | *P* | OR(95%CI) | *P* | OR(95%CI) | *P* |
| UA levels | 1.00(0.98-1.02) | 0.96 | 0.96(0.90-1.02) | 0.29 | 0.99(0.97-1.00) | 0.15 | 0.98(0.96-1.00) | 0.06 |

UA, uric acid; GDM, gestational diabetes mellitus; CI, confidence interval; MR, Mendelian randomization; IVW, inverse variance weighting.
